# Supplementary material for: Comparative Analysis of Posiphen Pharmacokinetics across Different Species—Similar Absorption and Metabolism in Mouse, Rat, Dog and Human
Source: Biomolecules. 2024 May 15;14(5):582. doi: 10.3390/biom14050582 (PMC11117716; doi:10.3390/biom14050582)
Supplement: Supplementary file 1 [file biomolecules-14-00582-s001.zip › Supplementary Material S2.pdf]

Supplementary Material S2

	CD-1 Mice, n=7			Sprague Dawley Rats, n=6			Beagle Dogs, n=12		
	Posiphen 65 mg/kg, plasma			Posiphen 40mg/kg, plasma			Posiphen 20mg/kg, plasma		
Time, h	Mean	SD	%CV	Mean	SD	%CV	Mean	SD	%CV
0.25	937	229	24.4	1090	724	66.7	2.34	7.95	339
0.5	939	445	445	1180	684	58.2	241	333	138
1	610	167	167	771	627	81.3	326	236	72.4
2	571	283	283	381	523	137	203	149	73.2
4	19.5	16.5	16.5	243	276	113	32.3	13.7	42.5
8	ND	ND	ND	234	188	80.3	4.4	2.23	50.7
12	ND	ND	ND	ND	ND	ND	1.27	0.678	53.5

Supplementary Table S1. Mean maximum concentration (Cmax), standard deviation (SD) and % of coefficient of variance (%CV) of Posiphen in mice, rats and dogs over a 12-hour period.

	CD-1 Mice, n=7					
	N1-norposiphen, plasma			N8-norposiphen, plasma		
Time, h	Mean	SD	%CV	Mean	SD	%CV
0.25	350	20.6	5.88	882	200	22.7
0.5	484	87.1	18.0	1309	81.6	6.24
1	446	140	31.3	1562	558	35.7
1.25	554	275	49.7	1793	868	48.4
1.5	525	279	53.0	1931	909	47.1
2	419	195	46.6	1826	869	47.6
4	83.6	28.3	33.8	478	189	39.5
8	3.64	ND	ND	11.3	9.66	85.2
12	0	ND	ND	0	ND	ND

Supplementary Table S2. Mean maximum concentration (Cmax), standard deviation (SD) and % of coefficient of variance (%CV) of N1- and N8-norposiphen in mice over a 12-hour period.
