# Supplementary material for: Comparative Analysis of Posiphen Pharmacokinetics across Different Species—Similar Absorption and Metabolism in Mouse, Rat, Dog and Human
Source: Biomolecules. 2024 May 15;14(5):582. doi: 10.3390/biom14050582 (PMC11117716; doi:10.3390/biom14050582)
Supplement: Supplementary file 1 [file biomolecules-14-00582-s001.zip › Supplementry Material S1.pdf]

Supplementary Material S1

Pharmacokinetic Data Analysis Methods

Structural Models

The structural models tested in this analysis were 1, 2, and 3-compartment models with either linear or nonlinear elimination.

General Pharmacokinetic Modeling Considerations

Modeling was performed using the First Order Conditional Estimation Method (FOCE). A log-transform both sides (LTBS) approach for the residual error model was implemented. Interaction is unnecessary because the residual error model is homoscedastic, and the interaction method is only relevant for proportional error models [**Error! Reference source not found.**].

The asymptotic standard errors (SEs) and nonparametric bootstrapped confidence intervals (CIs) estimated the parameter precision. Other model statistics were evaluated, such as etabar and associated p values.

The final basic structural model was selected based on the goodness-of-fit as judged by the change in the objective function (OBJ) and various diagnostic plots [predicted (PRED)/individual predicted (IPRED) versus observed concentrations, conditional weighted residuals [(C)WRES] /individual WRES (IWRES) versus time, CWRES /IWRES versus PRED/IPRED).

Statistical Model for Pharmacokinetic Inter-Individual Variation

Unexplained inter-individual variability (IIV) in structural model parameters was estimated using the following error model:

$$P_j = TVP \cdot e^{\eta_j}$$

In this equation, P_j is the individual value for the PK parameter [i.e., clearance (CL)] in the *jth* individual, and η_j is an independent random variable with a mean of zero and variance ω_P^2. This error model assumes a log-normal distribution for the P_j values. Estimates of inter-individual variance in P are presented as the square root of ω_P^2, which approximates the coefficient of variation for a log-normally distributed quantity.

Skewness and kurtosis of the eta distributions were evaluated graphically and did not show substantial skewness or kurtosis; therefore, transforms were not evaluated.

The highest feasible number of variance terms was included in the OMEGA matrix.

Statistical Model for Pharmacokinetic Residual Variation

Plasma concentration data were evaluated using an LTBS approach. In this case, the difference between model-predicted concentrations and observed concentrations was modeled with a proportional error model (additive on the log scale):

$$C_{ij} = \hat{C}_{ij} + \varepsilon_{2ij}$$

This approach allows simulations without the model producing negative concentration values and stabilizes model performance. Examples of potential sources of residual variability include assay error, incorrect model specification, and incorrect dose and/or sample records; however, procedural errors such as the latter two examples above may also be reflected in ω_P^2.

Covariate Models

The covariate models used in this analysis were defined to represent covariate influences as "shifts" in the parameter of interest from the parameter value observed in a hypothetical "reference" subject. The reference subject was defined as a subject with demographic factors equal to the median (weight, age, etc.) or most prevalent (sex, race, etc.) demographics in the dataset. Covariates were not centered due to the known issues with this procedure (e.g., the ability for parameter values to become negative when a subject covariate value is substantially lower than the reference value, generally less numerical instability than normalizing provides). However, covariates were normalized to the approximate median or a reference value (e.g., weight of 70 kg) as this avoids the issues associated with centering.

Continuous covariates, such as body surface area (BSA), were modeled using the general equation:

$$\mathrm{TVP} = P_{pop} \bullet \Pi_{i=1}^{n} \mathrm{cov}_i^{\theta_i}$$

Where TVP represents the model predicted PK parameter [apparent CL (CL/F), apparent central volume (V1/F), apparent intercompartmental clearance (Q/F), apparent peripheral volume (V2/F)] for the "typical" individual with covariate value(s) cov_i, P_{pop} represents the population central tendency for the PK parameter TVP, cov_i represents the individual value for the covariate normalized for the population means, and θ_i represents a scale factor. With this model type, if θ_i=0, the covariate's influence is dropped from the model; if θ_i=1, a directly proportional relationship is specified; and if θ_i is less than or greater than 1, a non-linear relationship is specified. Negative θ_i values specify a non-linear inverse relationship. Diagnostic plots were examined during the development of the model to assess the appropriateness of covariate models.

Categorical covariates, such as sex, were modeled using the general equation:

$$\mathrm{TVP} = P_{pop} \bullet \Pi_{i=1}^{n} \theta_i^{\mathrm{cov}_i}$$

In this equation, θ_i is a direct proportionality constant. With this model type, θ_i is fixed to 1 for the reference subgroup (i.e., males) and estimated for the test subgroup (i.e., females) since the values of the covariates will be 1 for reference and 0 for the test.

Covariates Assessed

The covariates listed in Table S1 were assessed for influence on the PK variability of posiphen.

Table S1 Planned Covariate Evaluations

Parameter	Covariates
Clearance	Age, Height, Bodyweight, Body Mass Index, Body Surface Area, Albumin, Alkaline Phosphatase, Alanine Aminotransferase, Aspartate Aminotransferase, Bilirubin, Creatinine Concentration, Creatinine Clearance, Hemoglobin, Platelets, Dose, Sex, Race, Subject Type, Ethnicity
Absorption Rate Constant	Fed State, Dose level, Race, Sex, Bodyweight
Relative Bioavailability	Fed State, Dose level
Inter-compartmental clearances (Q)	Age, Bodyweight, Body Mass Index, Body Surface Area, Sex, Subject Type
Volumes of Distributions	Age, Bodyweight, Body Mass Index, Body Surface Area, Sex, Subject Type

Model Selection Criteria

Decision-making during model building was guided by evaluating the change in the OBJ between model runs, evaluating the magnitude of inter-individual and residual variance, and examining residual diagnostic plots. The chi-squared test ($p<0.01$) for the log-likelihood difference in OBJ between nested models with degrees of freedom equal to the difference in the number of parameters between models was used to declare the superiority of one model over another. The $p<0.01$ corresponds to a reduction in OBJ of ≥ 6.64 when comparing models that differ by one parameter. The Akaike Information Criteria (AIC) was used to compare non-nested models and models with the same number of parameters.

The covariance step was implemented with each NONMEM run, and standard errors for parameter estimates and the correlation between parameters were evaluated. Base models that resulted in parameter estimates with high associated standard error (> 35% of the parameter estimate) and models with a high degree of correlation between parameters (>90%) were carefully evaluated and re-parameterized or possibly rejected. The superior model should have also had an associated reduction in the magnitude of inter-individual and/or residual variance estimates and improved residual plots.

Covariate analysis proceeded by separately examining the influence of each covariate alone on the base model. The p-value was used to rank the resulting covariate models for the likelihood ratio test (LRT) comparison with the base model (adjusted for the number of additional parameters in the covariate model). Those with a p-value less than 0.01 were considered in more detail. Those covariates that were identified singly were pooled in various combinations. The model with all candidate covariate relationships was declared the "full" model and subjected to a backward elimination process using a p-value of 0.005 for the LRT comparison. Therefore, a covariate was considered significant if the p-value for removing the covariate from the full model is less than 0.005, corresponding to a decrease in the OBJ by >7.9 points.

The final covariate model was chosen based on models with a statistically significant improvement in the objective function value after forward addition and backward deletion.

The model passed the covariance step and had a condition number less than 20, had precise estimates of the covariate parameter (asymptotic se% < 50%), and reduced the between-subject variability (BSV) of the associated population parameters to a clinically important extent (> 5% reduction in BSV).

Preliminary Evaluation of Final Model

The preliminary evaluation of model performance was based on the parameter estimate reliability and the stability of the model. The evaluations described below were conducted on the base (i.e., no covariates) and the final model (i.e., including covariates).

Model Stability

Where feasible, model stability was tested by evaluating the condition number. The condition number is calculated as the square root of the ratio of the largest to the smallest eigenvalue of the correlation matrix of the parameter estimates. Consequently, condition numbers can be calculated if the $COV step is completed successfully. The condition number is acceptable if it indicates that the omega matrix is well-conditioned. The condition number was used to ascertain the stability of parameter estimates. A condition number of less than 20 suggests that the degree of collinearity of the parameter estimates is acceptable. A condition number over 100 indicates that the model may be unstable due to high collinearity [**Error! Reference source not found.**]. In such cases, the model was simplified, the condition number re-computed, and the model re-evaluated.

The estimates of eta-bar and the associated p-values from the model output were recorded. An etabar value should be near 0, and the p-value should be near 1.

Finally, where feasible, symmetric 95% CIs were computed based on the asymptotic standard errors of the parameter estimates.

Parameter Shrinkage

One potential problem with population analysis is the tendency of the individual parameter estimates to "shrink" toward the mean value. This shrinkage is usually associated with too little data from each individual to provide a robust individual parameter estimate. When the individual parameter estimates exhibit substantial shrinkage, they no longer reflect the individual PK behavior, affecting derived parameter estimates such as the area under the concentration-time curve (AUC). Therefore, the extent of Bayesian shrinkage was assessed to evaluate the current database's ability to provide adequate individual parameter estimates for further derived parameter values. Inter-individual parameter shrinkage was evaluated before the inclusion of covariates into the model. Parameter shrinkage is a standard output from NONMEM. Small values for shrinkage (i.e., less than 30%) indicate good individual estimates of a parameter of interest, while large values would indicate poor individual estimates of a parameter of interest [**Error! Reference source not found.**]. In addition to the computation of shrinkage, frequency histograms or quantile-quantile (QQ) plots were constructed to visually assess the distribution of Bayesian estimates of the random effects.

Final Model Predictive Performance

Bootstrap Methods

If possible, the CIs of the parameters were taken from the asymptotic standard errors of the model. Bootstrapping was also used to evaluate parameter precision for final models.

Bootstrap methods are re-sampling techniques for assessing uncertainty. They are useful when inference is based on a complex procedure for which theoretical results are unavailable or to verify the usefulness of standard approximations for parametric models [**Error! Reference source not found.**]. Because bootstrapping allows the parameter values to converge independently, a reasonable estimate of the confidence intervals is generated. While asymptotic normality is a property of large sample analyses, the sample size is generally insufficient to justify this assumption in population pharmacometrics. Therefore, confidence intervals based on the standard errors of the typical values of parameter estimates can underestimate the uncertainties of these parameter estimates.

Five hundred bootstrap datasets were generated and run using the final model. The percentile bootstrap confidence intervals were constructed by taking the lower 5% and the upper 95% value of each parameter estimate from runs that converge successfully, as this interval should cover the true value of the parameter estimate approximately 90% of the time without imposing an assumption of symmetry on the distribution.

Visual Predictive Check

A VPC [**Error! Reference source not found.**] was conducted. Stratified VPCs based on dose level were constructed. For the final model evaluation, the 2.5th and 97.5th prediction intervals were constructed by simulating replicates of the dataset from which the model was developed. The observed data were then overlaid and compared to the prediction intervals. For the model to be acceptable, approximately 2.5% of the observed data should lie above the 97.5th prediction interval and 2.5% below the 2.5th prediction interval.

Final model parameters

Parameter (units)	THETA	THETA SE%
KA (1/hr)	7.36	29.8
ALAG1 (hr)	0.486	0.1
VMAX (ng/hr)	11600	4.7
KM (ng/mL)	13.3	11.6
V1 (L)	536	9.8
Q (L.hr)	57.1	6
V2 (L)	401	5.7
Residual Error	43.8%	1
Effect of Dose on Ka	0.472	29.7
Effect of Dose on Relative Bio	0.312	8.3
Effect of Female Sex on Ka	1.4	24
Effect of Female Sex on Km	1.33	7.4
Effect of BMI on V1	-1.51	26.2
Effect of Dose on Km	0.0967	55.5
FASTKA	-0.675	12.8

(a)

Between patient variability and model diagnostic

ETA	BSVKA	BSVVMAX	BSVKM	BSVV1	BSVQ	BSVV2
ETASD %CV	115.8	-	25.8	55	-	27
ETASD se%	8.2	-	15	8.3	-	3.8
ETA P-value	0.378	-	0.995	0.827	-	0.671
ETA shrink SD%	9.4	-	38.1	17.6	-	27
EBV shrink SD%	18	-	47.7	28.3	-	83.9
EPS shrink SD%	9.1					

(b)

Basic Goodness of Fit

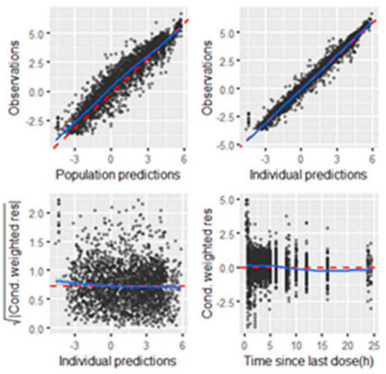

(c)

Visual Predictive Check

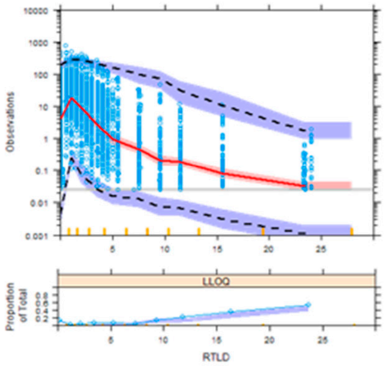

(d)

Figure S1. (**a**) Final model parameters; (**b**) between patient variability and model diagnostic; (**c**) Basic Goodness of Fit plots; (**d**) Visual Predictive Check.

References

1. Boeckmann, A.J.; Beal, S.L.; Sheiner, L.B. *NONMEM Users Guide—Parts I through VIII, NONMEM Installation Guide*; NONMEM Project Group; University of California: San Francisco, CA, USA, 1998.
2. Glantz, S.A.; Slinker, B.A. *Primer of Applied Regression and Analysis of Variance*; McGraw Hill: New York, NY, USA, 1990; p. 225.
3. Bergstrand, M.; Karlsson, M.O. Handling data below the limit of quantification in mixed effect models. *AAPS J.* **2009**, *11*, 371-380. https://doi.org/10.1208/s12248-009-9112-5. PMID: 19452283; PMCID: PMC2691472.
4. Yafune, A.; Ishiguro, M. Bootstrap approach for constructing confidence intervals for population pharmacokinetic parameters. I: A use of bootstrap standard error. *Stat. Med.* **1999**, *18*, 581–599.
5. Bruno, R.; Vivier, N.; Vergniol, J.C.; Phillips, S.; Montay, G.; Sheiner, L.B. A population pharmacokinetic model for docetaxel (taxotere): Model building and validation. *J. Pharmacokinet. Biopharm.* **1996**, *24*, 153–172.
