# Supplementary figures and images for: Comparative Analysis of Posiphen Pharmacokinetics across Different Species—Similar Absorption and Metabolism in Mouse, Rat, Dog and Human
Source: Biomolecules. 2024 May 15;14(5):582. doi: 10.3390/biom14050582 (PMC11117716; doi:10.3390/biom14050582)

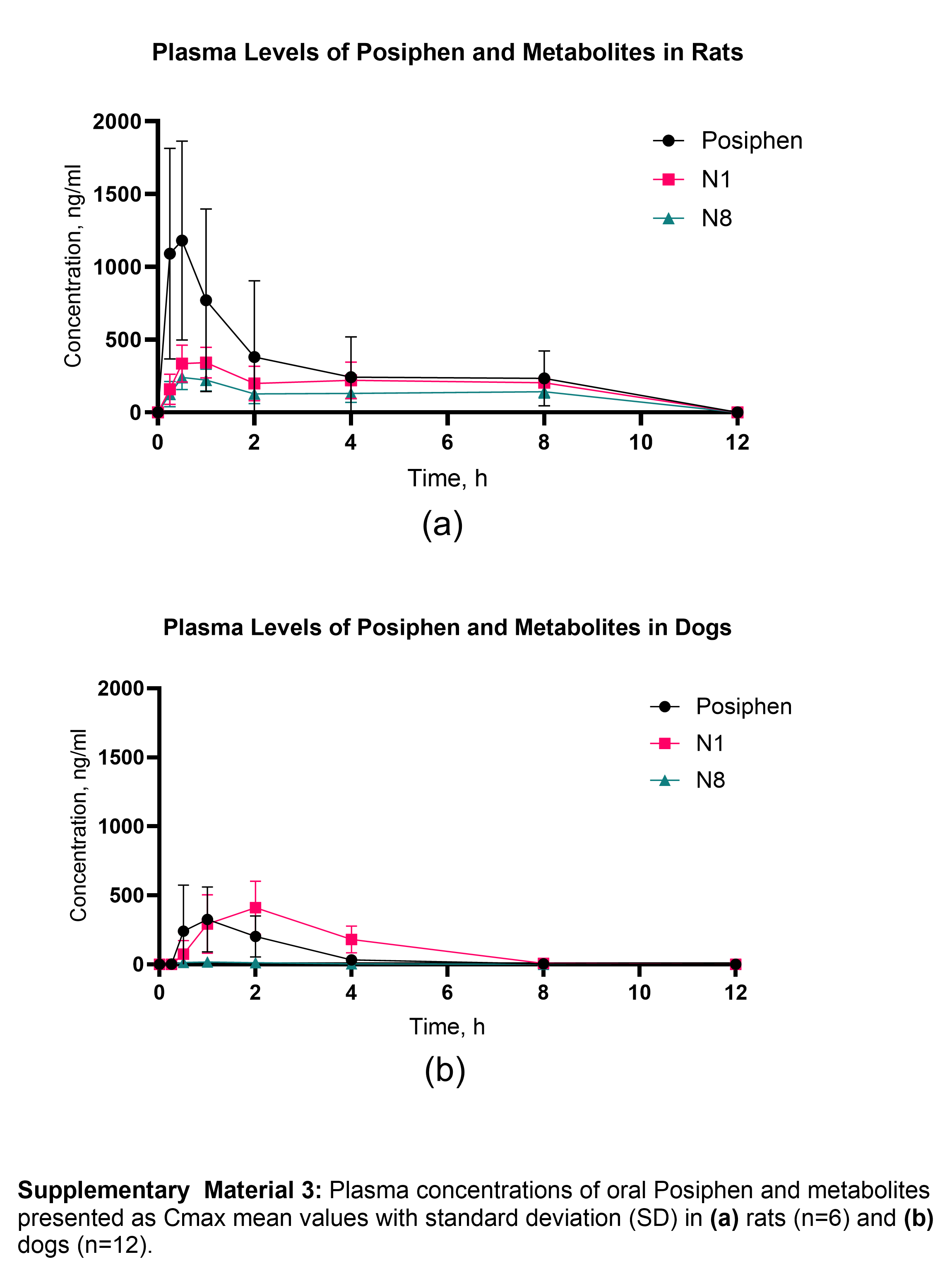

Supplement: Supplementary file 1 [file biomolecules-14-00582-s001.zip › Supplementary Figure S3.png]
